# Hemoglobin, oxygen transport reserve, and risk in hypertensive obstructive sleep apnea

**DOI:** 10.1038/s41371-026-01142-9

**Published:** 2026-04-02

**Authors:** Bülent Özlek

**Affiliations:** Muğla Sıtkı Koçman University, School of Medicine, Department of Cardiology, Muğla, Turkey

**Keywords:** Risk factors, Prognosis

## Abstract

Obstructive sleep apnea (OSA) and hypertension frequently coexist and together amplify cardiovascular risk, yet risk stratification in OSA remains largely centered on desaturation metrics. In this editorial, we contextualize the findings of Guo et al., who report an L-shaped association between admission hemoglobin levels and all-cause mortality among hospitalized patients with hypertensive OSA. We discuss the hypertensive OSA phenotype as a high-risk intersection of hypoxic and hemodynamic stress and emphasize arterial oxygen content—determined by both hemoglobin concentration and arterial oxygen saturation—as an integrative determinant of oxygen delivery reserve. Within this framework, reduced hemoglobin may further compromise tissue oxygen transport in an already vulnerable population. We underscore that hemoglobin should be interpreted as a risk modulator rather than a direct therapeutic target, and that causal inference cannot be drawn from observational data. Future prospective studies integrating polysomnographic metrics with longitudinal hemoglobin assessments are needed to clarify the clinical implications.

Obstructive sleep apnea (OSA) and hypertension frequently coexist and appear to exert mutually reinforcing effects on cardiovascular risk through mechanisms that include intermittent hypoxemia, autonomic instability, and adverse vascular remodeling [1, 2]. Contemporary risk assessment in OSA, however, remains largely centered on apnea burden and oxygen desaturation indices, implicitly treating arterial oxygen saturation as a sufficient surrogate for effective tissue oxygen delivery. In this context, Guo et al. explore a pragmatic and clinically accessible marker: among hospitalized patients with both OSA and hypertension, does admission hemoglobin stratify short- and long-term mortality risk? [3].

Using the MIMIC-IV critical care database, the investigators analyzed 6155 patients with a first hospitalization coded for both OSA and hypertension [3]. Hemoglobin measured within 24 h of admission was inversely associated with 365-day and longer-term all-cause mortality after multivariable adjustment. Mortality was highest in the lowest hemoglobin quartile and declined stepwise across higher quartiles. Importantly, the relationship was nonlinear, following an L-shaped pattern with an inflection around 11.9 g/dL. Below this threshold, mortality risk rose steeply as hemoglobin declined; above it, the association attenuated substantially [3]. These findings suggest that lower hemoglobin levels may mark a particularly vulnerable phenotype in hypertensive OSA, whereas higher hemoglobin levels confer diminishing prognostic discrimination once a threshold of physiological reserve is reached.

The biological rationale becomes clearer when framed in terms of oxygen content rather than saturation. Arterial oxygen content is commonly approximated as: CaO₂ = 1.34 × Hemoglobin × (SaO₂/100) + 0.003 × PaO₂ [4]. In OSA, recurrent nocturnal desaturation reduces SaO₂; anemia reduces hemoglobin. Together, these mechanisms may compound the reduction in arterial oxygen content, potentially translating similar saturation profiles into markedly different oxygen delivery to susceptible organs. This perspective also underscores a practical limitation of routine monitoring: pulse oximetry reflects hemoglobin saturation, not hemoglobin concentration, and therefore cannot detect reductions in oxygen-carrying capacity when hemoglobin is low [4]. Thus, seemingly acceptable oxygen saturation values do not necessarily imply preserved oxygen delivery reserve. This integrated conceptual model of the hypertensive OSA phenotype and oxygen transport reserve is illustrated in Fig. 1.

**Fig. 1:**
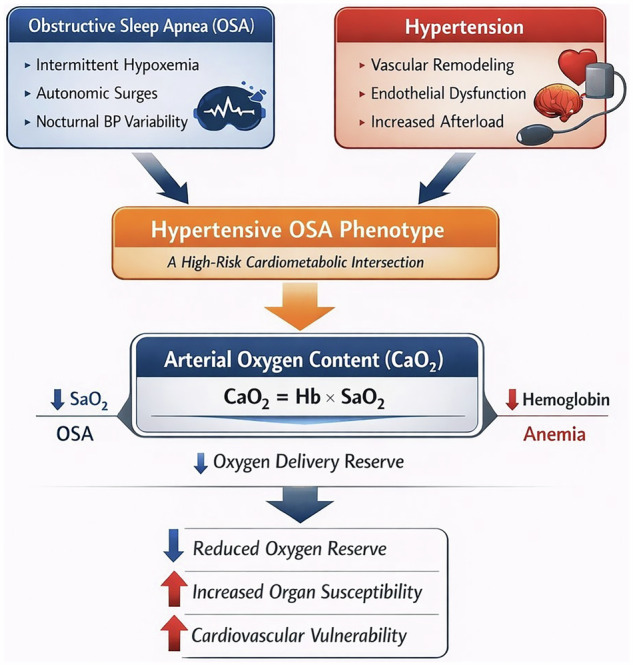
Conceptual framework of the hypertensive obstructive sleep apnea phenotype and the modulating role of oxygen transport reserve. Hb hemoglobin, SaO_2_ arterial oxygen saturation.

Beyond oxygen transport, hemoglobin may contribute to OSA-related hypertension through neurohumoral and vascular pathways. OSA is characterized by recurrent physiological stressors—including intermittent hypoxemia and autonomic surges—that drive sustained sympathetic activation and vascular dysfunction, thereby reinforcing hypertensive risk [1, 2]. Population-level data further suggest that anemia and OSA may interact in relation to hypertension risk [5], supporting a compounded biological phenotype rather than independent, parallel comorbidities. From a translational perspective, the erythropoietin (EPO) axis warrants caution. EPO-based therapies are known to influence blood pressure regulation [6], underscoring that anemia correction in hypertensive OSA should not be considered physiologically neutral.

Another point of interest is the L-shaped association reported by Guo et al. [3], in contrast to the U- or J-shaped hemoglobin–mortality relationships commonly described in broader populations [7]. In large screening cohorts, both low and high hemoglobin values have been linked to increased cardiovascular and all-cause mortality [7]. In hospitalized patients with OSA and hypertension, several factors may disproportionately accentuate left-tail risk. Intermittent hypoxemia may create a physiological context in which reductions in hemoglobin below accepted anemia thresholds markedly diminish oxygen content reserve, particularly in a population already exposed to recurrent desaturation [8]. Conversely, the distribution of hemoglobin values in this hospitalized cohort may not have included sufficient representation of extreme erythrocytosis to detect a clear right-tail hazard. Acute illness, inflammation, bleeding, and hemodilution—conditions more prevalent among hospitalized patients—may also cluster among those at highest risk, potentially amplifying the observed association. These explanations are not mutually exclusive and underscore the complexity of interpreting observational patterns.

From a clinical standpoint, the most defensible implication is enhanced risk stratification and structured evaluation rather than reflexive correction of hemoglobin levels. Hemoglobin is inexpensive, routinely measured, and available early in hospitalization. If replicated in cohorts with objective polysomnographic data, hemoglobin could serve as a pragmatic marker of oxygen transport reserve, complementing hypoxic burden metrics and established cardiovascular risk assessment [3, 4]. In practice, low hemoglobin in a hypertensive patient with suspected or confirmed OSA should prompt systematic evaluation for the underlying cause of anemia and careful optimization of comorbidity management. Foundational management principles remain paramount: appropriate evaluation and treatment of OSA and optimized antihypertensive therapy. Randomized evidence indicates that continuous positive airway pressure therapy can reduce ambulatory blood pressure in patients with resistant hypertension and OSA, although the magnitude of benefit is modest and heterogeneous [9].

Whether correcting anemia improves survival in hypertensive OSA remains uncertain. Observational associations cannot establish causality, and therapeutic strategies entail potential trade-offs. EPO-based interventions may elevate blood pressure [6], and excessive hemoglobin correction could, in theory, shift patients into ranges associated with increased risk in general population analyses [7]. A prudent translational approach would prioritize cause-directed anemia management integrated with optimized OSA and hypertension care, while reserving mortality-reduction claims for future randomized trials.

The study by Guo et al. [3] also highlights important research priorities. OSA ascertainment based on administrative coding may lead to misclassification, and the absence of polysomnographic metrics—such as the apnea–hypopnea index, oxygen desaturation index, or time spent below 90% saturation—precludes direct evaluation of whether hemoglobin modifies the effect of nocturnal hypoxic burden on mortality [3]. A single admission hemoglobin measurement may not reflect a stable physiological status. Prospective studies integrating detailed sleep phenotyping, serial hemoglobin indices, and oxygen content modeling are needed to quantify cumulative hypoxic burden. Such data could inform targeted interventional trials in well-characterized subgroups.

In summary, hemoglobin should be viewed not merely as a laboratory parameter but as a determinant of arterial oxygen content [4]. In hypertensive OSA, lower hemoglobin appears to identify a subgroup in which risk clusters around an anemia-range threshold [3, 8]. Pending causal evidence, hemoglobin may reasonably inform prognostic assessment and diagnostic evaluation. However, therapeutic extrapolation toward “hemoglobin correction to improve survival” should remain measured, physiologically informed, and attentive to the hemodynamic implications of erythropoiesis-stimulating strategies [6].
